# Editorial: Towards Users' Optimal and Pleasurable Experience in Smart Environments

**DOI:** 10.3389/fpsyg.2020.583663

**Published:** 2020-10-20

**Authors:** Mi Jeong Kim, Xiangyu Wang, Inhan Kim

**Affiliations:** ^1^School of Architecture, Hanyang University, Seoul, South Korea; ^2^Australasian Joint Research Centre for Building Information Modelling, Curtin University, Bentley, WA, Australia; ^3^Department of Architecture, Kyung Hee University, Yongin, South Korea

**Keywords:** smart environment, human factors, optimal experience, pleasurable interaction, human-computer interaction

## Introduction

The transition from the pre-digital to the digital age has been acknowledged as a fundamental step that changed people-environment relations in contemporary society, with relevant implications and important psychological outcomes in our daily lives (Stokols, 2018). Smart environments are one of the key elements that characterize such transition. The smart environment that incorporates interactivity is an important research theme in the architectural domain. Consequently, the challenges involved in developing intelligent environments that can support a comfortable lifestyle have attracted extensive research attention. Living spaces in which a wide range of smart devices are integrated into building components can support occupants' activities and extend their capabilities. Such integration has affected occupants' cognitive experiences related to their surrounding environments. To provide readers, a view of recent developments in this research area, this special issue focuses on the application of a human-centered approach in the design of smart environments ensuring occupants' optimal and pleasurable experiences. After a rigorous review by experts on the submitted articles, 12 papers are included in this special issue. These articles can be organized into three categories as follows.

## Conceptual Models and Scenarios For Smart Environments Emphasizing Experience

In implementation of smart environments, the conceptual models are essential to identify the factors critical to ensuring optimal interactivity, as well as the potential scenarios that effectively create pleasurable experiences. To this end, Kim and Maher presented three conceptual metaphors for designing smart environments—device, robot, and friend—to frame new ways for the interaction design of smart environments. The metaphorical design framework can support designers in creating novel interaction experiences by facilitating the formation of a common mental model for new interactive designs. Further, Kim et al. investigated the ways to control and adapt technology to fulfill the daily needs of users and developed a framework of smart home services from the perspective of supporting user experience. Since the success of smart homes fundamentally depends on their adoption and use by people in the context of daily life, they developed design solutions for smart homes through user-centered scenarios. Moreover, Lee and Park proposed an immersive experience service model for elderly welfare centers by analyzing the health benefits of immersive experience technologies and related services. They showed that the use of these technologies, such as virtual reality and mixed reality, could mitigate physical, and spatial constraints by immersing users into the desired environment and would thus contribute substantially to maintaining the health of the elderly. Interestingly, the potential of smart environments is not limited to the architectural domain but is applied to the clothing area in terms of interfaces to smart spaces. From this perspective, Yoo et al. argued that clothing combined with digital technology can be a wearable interface that adapts to environmental changes and increases biological limits as an emotional communication space. They proposed a conceptual biomorphic clothing sculpture interface, as a knowledge base that using the parametric design methodology from the 3D form development of architecture.

## Proposing Smart Home, Facility, Industry, and City Based on Users' Needs

Several types of smart environments are available for areas such as the home, a facility, an industry, and a city. To implement pleasurable smart environments, users' needs should be identified and understood in detail not Only from a developer or a designer's perspective but ALSO from that of those individuals. For this purpose, Lee and Kim critically reviewed smart homes for older adults using an evaluation framework comprising four categories: wellness, independence, acceptance, and design. They asserted that it is important to understand older adults' cognitive and emotional aspects and not just emphasize the efficiency of smart homes. Unlike the general studies on university facilities that have been focused mainly on space management, Kim and Kim proposed an effective university management plan that reflects the needs of the main users—students—to improve their satisfaction, which would lead to the optimal smart environment for them. Further, by focusing on newly developed intelligent devices for assisting elderly people in China, Meng et al. investigated the opportunities and the challenges for the country's elderly care industry in offering a smart environment based on occupants' needs and preferences. Their findings may enable industry stakeholders to make better decisions regarding smart elderly care services. Lastly, An et al. proposed a needs map for service design in smart cities, which can be applied to various stages of service development, since they consider the inclusion of actual service users in the service design a key factor in determining the sustainability of services for smart cities. They demonstrated the feasibility of the proposed needs map by applying it to the service design for the smart city experience zone in Daejeon, Korea.

## Empirical Studies Focusing on Interactivity and Engagement

Researchers often conduct empirical studies to explore the effects of variables on specific systems or to identify the practicability of proposed ideas in defined areas. Thus, empirical studies that focus on the interactivity and the engagement in smart environments should be performed to ensure that the user experience is optimal and pleasurable. In this regard, Angioletti et al. adopted a neuroscientific multimethodological approach to define the possible effects of smart home systems on user experience and provided evidence for neurophysiological correlates of user experience in such systems. Their study forms the basis for understanding users' responsiveness of these systems in terms of their cognitive and emotional engagement while interacting with a complex system. In addition, Cho and Suh explored spatial color efficacy in an eye-tracking study by examining different applications of the same color combination in a retail interior environment to determine whether these cause different emotional responses, which, in turn, influence viewers' perception of luxury and their intention to stay. Their results provide useful guidelines to designers in planning retail stores to achieve the desirable level of interactivity with users. Similarly, Kim and Kim conducted an eye-tracking experiment to evaluate the effects of background music on the perceived atmosphere of a service setting. Their findings indicate that the differences in scan paths and locations between slow tempo music and fast tempo music change over time. Further, Han et al. focused on smart accessibility to develop a design process of a model that integrates geospatial data from various sources to present user-customized universal design information. They emphasized that the process of communication with users is a key issue and that providing information services using a geospatial database is as important as improving physical accessibility.

## Toward Pleasurable, Optimal Smart Environments

This Research Topic specifically focuses on research and case studies that illustrate opportunities and challenges related to human-environment interactive experiences enabled through smart services and technologies. This collection contains new studies that provide cognitive insights on understanding occupants' needs and experiences which might motivate others in this field to develop new cognitive paradigms. All the proposed methods, models, scenarios, and applications in these studies contribute to the current understanding of the smart environment paradigm and are likely to inspire further research on addressing the challenges related to creating pleasurable, optimal smart environments.

## Author Contributions

All authors listed have made a substantial, direct and intellectual contribution to the work, and approved it for publication.

## Conflict of Interest

The authors declare that the research was conducted in the absence of any commercial or financial relationships that could be construed as a potential conflict of interest.
